# An Advanced Hybrid Technique of DCS and JSRC for Telemonitoring of Multi-Sensor Gait Pattern

**DOI:** 10.3390/s17122764

**Published:** 2017-11-29

**Authors:** Jianning Wu, Jiajing Wang, Yun Ling, Haidong Xu

**Affiliations:** College of Mathematics and Informatics, Fujian Normal University, Fuzhou 350117, China; jiajingwang_1993@126.com (J.W.); yunling93@163.com (Y.L.); sihaijunmi@fjnu.edu.cn (H.X.)

**Keywords:** multi-sensor gait classification, distributed compressed sensing, joint sparse representation classification, telemonitoring of gait

## Abstract

The jointly quantitative analysis of multi-sensor gait data for the best gait-classification performance has been a challenging endeavor in wireless body area networks (WBANs)-based gait telemonitoring applications. In this study, based on the joint sparsity of data, we proposed an advanced hybrid technique of distributed compressed sensing (DCS) and joint sparse representation classification (JSRC) for multi-sensor gait classification. Firstly, the DCS technique is utilized to simultaneously compress multi-sensor gait data for capturing spatio-temporal correlation information about gait while the energy efficiency of the sensors is available. Then, the jointly compressed gait data are directly used to develop a novel neighboring sample-based JSRC model by defining the sparse representation coefficients-inducing criterion (SRCC), in order to yield the best classification performance as well as a lower computational time cost. The multi-sensor gait data were selected from an open wearable action recognition database (WARD) to validate the feasibility of our proposed method. The results showed that when the comparison ratio and the number of neighboring samples are selected as 70% and 40%, respectively, the best accuracy (95%) can be reached while the lowest computational time spends only 60 ms. Moreover, the best accuracy and the computational time can increase by 5% and decrease by 40 ms, respectively, when compared with the traditional JSRC techniques. Our proposed hybrid technique can take advantage of the joint sparsity of data for jointly processing multi-sensor gait data, which greatly contributes to the best gait-classification performance. This has great potential for energy-efficient telemonitoring of multi-sensor gait.

## 1. Introduction

Recently, wireless body area networks (WBANs), as an emerging wireless sensor networks technology that is applied to the human body [1,2,3], has been more and more popular in gait telemonitoring applications [4,5,6]. In such WBANs, each sensor node usually takes the form of a small wearable device equipped with a microcontroller, accelerometer, gyroscope, wireless communication module, and battery. These wearable sensors are co-located on the body for acquiring gait data of people who freely walk in a home or outdoor setting. Moreover, all acquired data can be transmitted, via internet, to a remote terminal such as a hospital for gait monitoring or assessment by further data processing such as gait classification. This greatly contributes to the telemonitoring of gait pattern change, and people do not often visit a hospital for gait function assessment [4,5,6,7]. However, in clinical applications, continuous acquisition of the multi-sensor gait data over the long term is usually required for further data processing, in order to accurately evaluate the gait pattern change [8,9,10]. In such a case, there have been several challenging existing issues such as improving the energy efficiency of the sensor, getting the best classification performance, and getting a lower computation complexity. The above existing issues are strangling the WBANs-based gait telemonitoring application, and some advanced techniques for multi-sensor gait data analysis are urgently needed to tackle these existing issues [9,10,11,12].

As we know, due to the limited life of the battery, the wearable sensors are usually required to consume the least possible amount of energy for the continuous telemonitoring of gait [10,11,12,13]. How to improve the energy efficiency of the sensors has been one challenging endeavor in the WBANs-based telemonitoring applications [10,11,12]. Many previous studies found that most of the energy of the sensors is consumed by wireless communication, and their works mainly focused on developing the energy-aware MAC protocols for the low energy consumption of sensors. However, these protocols don’t provide reliable data transfer because heavy collision is usually generated by some adjacent sensors [10,12]. In recent ten years, compressed sensing (CS)—an emerging data compression methodology that data compression and reconstruction can be perfectly performed based on data sparsity—has attracted wide attention in the study of energy-efficient gait telemonitoring applications [14]. The basic idea is that a larger amount of the acquired gait data is firstly compressed on sensors, in order to significantly reduce the energy consumption during data transmission. Then, all compressed data received in the remote terminal are perfectly reconstructed to perform further data processing [15,16]. For example, Wu et al. investigated the application of CS for the energy-efficient telemonitoring of gait using acceleration data [16]. Although significant research efforts are made, many existing works have only showed that the CS technique for energy-efficient gait telemonitoring is feasible only using single-sensor data. Theoretically, the CS technique has no ability to simultaneously compress multi-sensor data for the energy efficiency of sensors. This leads to the possible loss of the highly-correlated information regarding the intrinsic dynamics of human movement. It is essential to search for an advanced technique for jointly processing multi-sensor gait data.

Further data processing such as gait classification is also very important for gait monitoring. How to develop the best gait-classification model has been another challenging issue in gait telemonitoring applications [16,17,18]. Some studies have showed that the original gait data are directly used to develop the gait-classification models based on machine learning algorithms. In these studies, some commonly used traditional machine learning algorithms include artificial neural networks (ANN) [18], decision tree, *k*-nearest neighbor (KNN) [17], support vector machine (SVM) [16,19], and Hidden Markov Model (HMM) [20,21,22]. Comparing these methods, the HMM technique has shown higher performance in gait pattern classification using on-body wearable sensors. For instance, Mannini et al. investigated the feasibility of human physical activity classification by using on-body wearable sensors. Especially, Taborri et al. proposed a novel HMM-based distributed classifier for the detection of gait phases by using a wearable inertial sensor network. In their proposed algorithm, a distributed stochastic model and a hierarchical-weighted classification are implemented to detect gait phases by simultaneously processing the data of multi-sensors placed on different body segments of lower limbs. Recently, an emerging sparse representation classification (SRC) algorithm has widely attracted the focus of gait classification. Its basic idea is that all gait training samples are directly used to construct an over-complete dictionary, and a test gait can be sparsely represented as a linear combination of just those training samples with same gait class. The residual error criterion is defined by solving sparse representation coefficients, in order to exactly determine the class of the test gait [23,24,25]. Zhang et al. investigated the feasibility of a sparse representation-based gait-classification model using wearable sensor gait data [24]. Also, Allen et al. studied the practicality of the sparse representation-based gait-classification model using wearable multi-sensor gait data [25]. Although the SRC algorithm can produce a better accuracy, it has a higher computational time cost. More importantly, all above studies don’t take into account the use of the gait data received at a remote terminal to develop a gait-classification model [26,27].

At the same time, other recent studies have focused on using the received gait data to develop gait-classification models. In view of the energy efficiency of sensors, recent relevant studies mainly concentrated on the combination of the CS technique with machine learning algorithms for gait classification. Their basic idea is that all compressed gait data received at a remote terminal are reconstructed by the CS technique, and the reconstructed gait data are then employed to develop gait-classification models based on machine learning algorithms [14,16]. In such works, one important step is to reconstruct gait data with a higher quality. However, the traditional CS technique has no ability to perfectly reconstruct the gait data; this is because gait data such as acceleration data are poorly sparse [14,16]. This possibly deteriorates the further gait-classification performance. Moreover, these hybrid techniques possibly produce a higher computational complexity. This motivates us to search for advanced approaches to the energy-efficient telemonitoring of multi-sensor gait [28,29].

It is well known that multi-sensor gait data generally interact in a complex fashion, an observation attributable to the intrinsic dynamics of human gait. Theoretically, we can assume multi-sensor gait data as a data ensemble with joint sparsity, in order to jointly process multi-sensors and thus capture the higher-correlation information associated with gait. This can potentially allow the best classification performance and energy efficiency of sensors. Recently, there have emerged some advanced techniques that have a superior ability to jointly process multi-signals based on data joint sparsity. Successful examples include distributed compressed sensing (DCS) [30,31] and the joint sparse representation classification (JSRC) model [32,33,34]. These two techniques have been widely applied in video coding [35,36,37], image fusion [38], and multichannel physiological monitoring [39]. So far, no studies have been reported where DCS and JSRC have been employed for jointly processing multi-sensor gait data.

In this study, a novel advanced hybrid technique of DCS and JSRC for the telemonitoring of multi-sensor gait is proposed based on data joint sparsity. Unlike the recent relevant studies, the advantage of our proposed technique is that we directly take advantage of the multi-sensor gait data compressed by DCS to develop the novel neighboring JSRC gait-classification models with better performance. This can not only avoid the complicated step of joint reconstruction of multi-sensor gait data, but can also produce the best classification performance as well as a lower computational time. In our proposed technique, the DCS technique is firstly utilized to simultaneously compress multi-sensor gait data, in order to potentially gain higher-correlation information as well as improved energy efficiency of the sensors. Then, all jointly compressed gait data are directly used to develop a novel neighboring sample-based JSRC model by defining the sparse representation coefficients-inducing criterion (SRCC), not to perform the reconstruction task. The test gait can be sparsely represented by constructing a new over-complete dictionary that includes a few local neighboring training samples containing more valuable information. This possibly produces the best classification performance as well as a lower computational time. Our proposed technique has great potential to enable continuous collection of people’s gait information and accurate human gait measurement anywhere and at any time. This possibly provides benefits in telemedicine applications such as the early identification of at-risk gait of elderly in the community, as well as the monitoring of gait rehabilitation progress of people at home. The multi-sensor gait data are selected from an open wearable action recognition database (WARD), in order to validate the feasibility of our proposed method. The results show the superior classification performance of our proposed techniques to the traditional techniques such as SRC and JSRC. Moreover, a lower computational time cost is spent in our proposed technique.

The rest of the paper is organized as follows. In Section 2, we describe the DCS technique for multi-sensor gait data. A novel neighboring JSRC model for multi-sensor gait classification is presented in Section 3. In Section 4, we describe the materials and methods for evaluating our proposed technique. The evaluation results obtained from multi-sensor gait data are given in Section 5. Discussion and conclusions are presented in Section 6 and Section 7, respectively.

## 2. Distributed Compressed Sensing for Multi-Sensor Gait Data

Theoretically, the DCS technique combines distributed source coding theory with compressed sensing theory [14,30,31]. It can take advantage of data joint sparsity to capture both inter- and intra-signal correlation among multi-sensor signals. In the DCS technique, three different joint sparsity models (JSM), such as JSM-1, JSM-2, and JSM-3 [30,31], are usually used to perform the simultaneous compression and the joint reconstruction task in the multi-signal case. In this study, since each sensor can acquire the same gait data, such as acceleration data, we assume that each sensor’s data has the same sparsity pattern but with different coefficients. Here, JSM-2 is adopted to jointly process the multi-sensor gait data [30,31]. That is, all J sensors’ gait data in WBANs are assumed as a data ensemble $X={\left[x_{1},x_{2},\cdots ,x_{J}\right]}^{T}\in R^{JN\times 1}$, where N denotes the length of each sensor data $x_{j}\in R^{N\times 1}(j=1,2,\cdots ,J)$. Here, we assume that each sensor data $x_{j}$ is sparsely represented as

(1) $$x_{j}=\Psi \theta_{j}$$

where Ψ denotes dictionary matrix vectors and $\theta_{j}$ is the corresponding sparse vector that satisfies the same support set Ω⊂{1,2,⋯,N}. Then, the multi-sensor gait data ensemble X can be represented as

(2) X=Ψθ

where $\theta ={\left[\theta_{1},\theta_{2},\cdots ,\theta_{j},\theta_{J}\right]}^{T}$ is the joint sparse coefficients vector and $\Psi =\left[\begin{array}{l} \Psi ,0,0,\cdots ,0\\ 0,\Psi ,0,\cdots ,0\\ \cdot \\ 0,0,0,\cdots ,\Psi \end{array}\right]$ is the corresponding dictionary matrix. Then, the multi-sensor gait data ensemble X can be rewritten as

(3) $${\left[x_{1},x_{2},\cdots ,x_{j},\cdots ,x_{J}\right]}^{T}=\left[\begin{array}{l} \Psi ,0,0,\cdots ,0\\ 0,\Psi ,0,\cdots ,0\\ \cdot \\ 0,0,0,\cdots ,\Psi \end{array}\right]{\left[\theta_{1},\theta_{2},\cdots ,\theta_{j},\cdots ,\theta_{J}\right]}^{T}.$$

Next, we define a measurement matrix $\Phi \in R^{{\mathrm{JM}}_{j}\times \mathrm{JN}}(\Phi_{j}\in R^{M_{j}\times N},M_{j}\ll N)$ as

(4) $$\Phi =\left(\begin{array}{llllll} \Phi_{1} & 0 & 0 & 0 & \cdots & 0\\ 0 & \Phi_{2} & 0 & 0 & \cdots & 0\\ \vdots & \vdots & \vdots & \vdots & \cdots & \vdots \\ 0 & 0 & 0 & 0 & \cdots & \Phi_{J} \end{array}\right).$$

Thus, the multi-sensor gait data ensemble X can be simultaneously compressed as

(5) $$Y=\Phi X=\left(\begin{array}{llllll} \Phi_{1} & 0 & 0 & 0 & \cdots & 0\\ 0 & \Phi_{2} & 0 & 0 & \cdots & 0\\ \vdots & \vdots & \vdots & \vdots & \cdots & \vdots \\ 0 & 0 & 0 & 0 & \cdots & \Phi_{J} \end{array}\right){\left[x_{1},x_{2},\cdots ,x_{j},\cdots ,x_{J}\right]}^{T}$$

where $Y={\left[y_{1},y_{2},\cdots ,y_{j},\cdots ,y_{L}\right]}^{T}\in R^{JM\times 1}$ is the jointly compressed multi-sensor data. Here, it is noted that each $y_{j}=\Phi_{j}x_{j},j\in \{1,2,\cdots ,J\}$ is performed independently of the others.

In the DCS technique, joint reconstruction is another important task. Its basic idea is to obtain an accurate solution of the joint sparse representation coefficients $\theta ={\left[\theta_{1},\theta_{2},\cdots ,\theta_{j},\theta_{J}\right]}^{T}$ by solving the following $l_{1}$-norm minimization:

(6) $$\hat{\theta }=\underset{\theta }{\mathrm{argmin}}||\theta |{|}_{l_{1}},s.t.\;Y=\Phi X.$$

Then, the joint reconstruction of multi-sensor data can be estimated as $\hat{X}=\Psi \hat{\theta }$. The commonly used joint reconstruction algorithms include the One-Step Greedy Algorithm (OSGA) [32] and the Simultaneous Orthogonal Matching Pursuit (SOMP) [31]. However, since multi-sensor gait data such as accelerometer and gyroscope data are poorly sparse, it is very difficult to guarantee the best solution of joint representation coefficients $\hat{\theta }$ by Equation (6). In fact, the jointly compressed multi-sensors data Y by DCS possibly contain enough highly-correlated discriminative information associated with gait. Theoretically, the compressed multi-sensor data Y can be directly used to develop a JSRC-based classification model, but not to perform a joint reconstruction task. This helps to improve multi-sensor gait-classification performance.

## 3. A Novel Neighboring JSRC Model for Gait Classification

**Figure 1:**
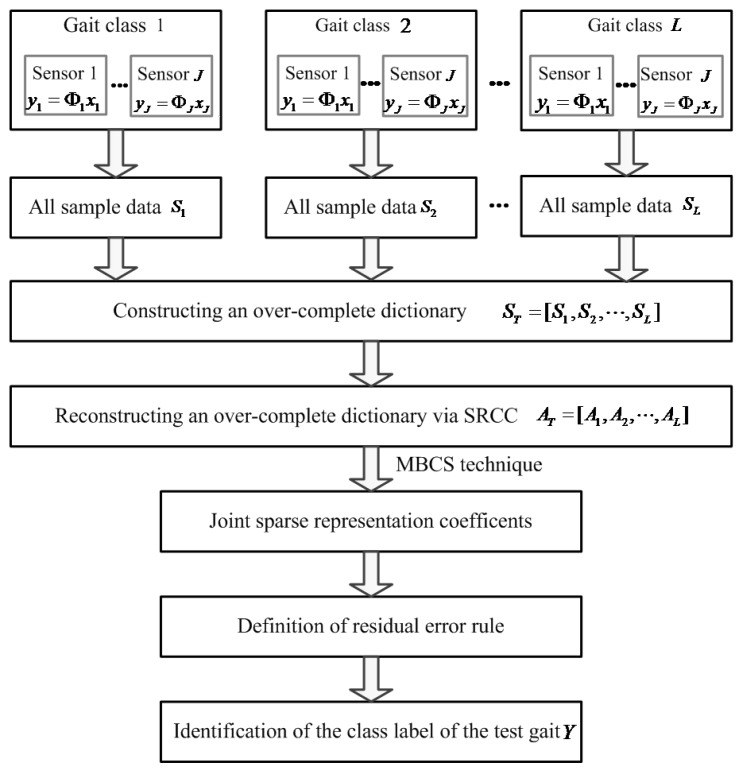
The block diagram of the proposed novel neighboring joint sparse representation classification (JSRC) model.

Theoretically, the JSRC model is used to generalize the traditional SRC model to the multi-signal case [33,40,41,42,43]. Its basic idea is to explore the joint sparse representation for the multiple input signals in the classification task, and the joint sparsity can be enforced by imposing a joint sparsity-inducing norm(such as $l_{1,2}$-norm, $l_{1,\infty }$-norm) penalty on representation coefficients [23,24,25]. However, due to the large-scale gait training samples in this study, more computational time and higher memory cost are needed for the matrix inverse operation in the JSRC algorithm. This possibly yields a high computational complexity as well as a poor classification performance. It is essential to search for a few training samples to develop the best JSRC-based gait-classification models. In this study, a novel neighboring JSRC model is developed based on a few neighboring training samples selected by the sparse representation coefficients-inducing criterion (SRCC). As we know, the same gait class can be theoretically spanned by the training samples (i.e., over-complete dictionary atoms) of the same class in a low-dimensional subspace [33,41,42,43,44,45,46,47,48]. The spanned training sample can be sparsely represented in the high-dimension space. Those samples with high values of sparse representation coefficients are selected as the nearest neighbor samples because they possibly contain the more valuable information associated with gait pattern change. So, these selected neighboring samples are used to reconstruct a new over-complete dictionary for multi-sensor gait classification. The block diagram of the proposed novel neighboring JSRC model is shown in Figure 1. In order to clearly describe the novel neighboring JSRC model, we firstly describe the construction of an over-complete dictionary using all original training samples.

### 3.1. Constructing an Over-Complete Dictionary for JSRC

In this study, we assume that each sensor node is equipped with a triaxial accelerometer (x,y,z) and a biaxial gyroscope (θ,ρ) [24,25], in order to capture more valuable information associated with gait. Firstly, we define a vector $a_{j}$ to denote the gait data of each sensor j(j=1,2,⋯,J) at time t, i.e.,

(7) $$a_{j}={\left(x_{j}\left(t\right),y_{j}\left(t\right),z_{j}\left(t\right),\theta_{j}\left(t\right),\rho_{j}\left(t\right)\right)}^{T}\in R^{5}.$$

Then, a vector a(t) is defined to represent multi-sensor gait data of all J sensors at time t, i.e.,

(8) $$a\left(t\right)={\left(a_{1}^{T}(t),a_{2}^{T}(t),\cdots ,a_{J}^{T}(t)\right)}^{T}\in R^{5J}.$$

Next, based on Equation (6), a matrix S is defined to represent the multi-sensor gait data ensemble during the length of time h:

(9) $$S={\left[a(1),a(2),\cdots ,a(h)\right]}^{T}={\left(s^{1},s^{2},\cdots {,s}^{J}\right)}^{T}\in R^{5J\times h}$$

where the sub-vector $s^{j}$ denotes the gait data of sensor j with duration h, $s^{j}={\left(a_{j}(1),a_{j}(2),\cdots ,a_{j}(h)\right)}^{T}\in R^{5\times h}$.

Here, assuming that there are L different gait classes, we can define a new matrix $S_{T}$ that concatenates the training sample from all L gait classes:

(10) $$S_{T}=\left[S_{1},S_{2},\cdots ,S_{i},S_{L}\right]\left(i=1,2,\cdots ,L\right)$$

where sub-matrix $S_{i}=\left(s_{i,1},s_{i,2},\cdots ,s_{{i,n}_{i}}\right)$ represents all training sample data of gait class i, and $n_{i}$ denotes the total of training samples of class i. In such a case, we can employ the matrix $S_{T}$ to construct an over-complete dictionary that consists of L sub-dictionary $S_{i}$ with respect to all L classes [23,25,33,41,43]. Thus, based on the constructed over-complete dictionary $S_{T}$, a test gait Y ($Y={\left[y_{1},y_{2},\cdots ,y_{j},\cdots ,y_{J}\right]}^{T}\in R^{JM\times 1}$) with class i can be joint-sparsely represented as

(11) $$Y=S_{T}W$$

where $W=\left[0,0,\cdots ,0,w_{i,1},w_{i,2},\cdots ,w_{i,n_{i}},0,\cdots ,0\right]$ denotes the joint sparse representation coefficients matrix whose entries are zero except those associated with gait class i, and the total number of samples $n=\sum_{i}^{L}n_{i}$.

Usually, the joint sparse coefficients matrix W can be estimated by solving the following joint sparse optimization problem [33,41,43]:

(12) $$\hat{W}=\arg\min||Y-S_{T}W|{|}_{F}^{2}\;s.t.\;||W{\left|\right|}_{l_{0}\backslash l_{2}}\leq k$$

where $||\cdot |{|}_{F}$ denotes the Frobenius norm, $\;||W{\left|\right|}_{l_{0}\backslash l_{2}}$ refers to the mixed norm that can be phrased as performing the $l_{2}$-norm cross the column and then the $l_{0}$-norm along the row, i.e., $||W|{|}_{l_{0}\backslash l_{2}}=||[||W(1,:)|{|}_{l_{2}},||W(2,:)|{|}_{l_{2}},\cdots ,||W(L,:)|{|}_{l_{2}}]|{|}_{l_{0}}$. k denotes the maximum number of the nonzero coefficients in W.

In fact, because the $l_{2}{/l}_{0}$ regularization norm needs the shared gait pattern across observation, the computation complexity reaches $O(w^{2}n)$ for the optimization solution of non-zero sparse coefficients w. That is, if large-scale training sample data are directly used to construct the over-complete dictionary, then a higher computational complexity is possibly yielded in estimating the optimization solution of w. Therefore, it is necessary to find a few training samples to reconstruct an over-complete dictionary for developing the JSRC-based multi-sensor gait-classification model with high quality.

### 3.2. Reconstructing a New Over-Complete Dictionary for JSRC

Here, the sparse representation coefficients-inducing criterion (SRCC) is used to find a few nearest neighboring samples containing the most valuable information, in order to reconstruct a new over-complete dictionary for JSRC. The detailed procedure of selecting the nearest neighbor samples is presented as follows.

**Step 1** The test sample $y_{j}$ from sensor j is linearly represented as

(13) $$y_{j}=w_{i,1}s_{1}^{j}+w_{i,2}s_{2}^{j}+\cdots +w_{{i,n}_{i}}s_{n_{i}}^{j}.$$

Then, the sparse representation coefficients w are obtained by solving the following $l_{2}$-minimization problem:

(14) $$\hat{w}=\underset{\hat{w}}{\arg\min}{\left||y_{j}-s^{j}w|\right|}_{2}+{\lambda ||w||}_{2}$$

where λ is a positive regularization, and the $l_{2}$-norm is defined as ${\left||w|\right|}_{2}={\sum_{i=1}^{n_{i}}\left|w_{i}\right|}_{2}$.

**Step 2** Based on the solution of w, the m training samples with the larger sparse coefficients values are selected as the nearest neighbor training samples. For gait class i, a new training sample set is defined as

(15) $$A_{i}=\left(s_{i,1},s_{i,2},\cdots ,s_{i,m}\right).$$

**Step 3** Based on Equation (15), a new over-completed dictionary is reconstructed as

(16) $$A_{T}={\left[A_{1},A_{2},\cdots ,A_{L}\right]}^{T}.$$

Therefore, a test gait Y with class i can be joint-sparsely represented as

(17) $$Y=A_{T}W.$$

Here, the joint sparse coefficients matrix W in Equation (17) can be estimated by solving the following $l_{2}{/l}_{0}$-minimization problem:

(18) $$\hat{W}=\arg\min||Y-AW|{|}_{F}^{2}\;s.t.\;||W{\left|\right|}_{l_{0}l_{2}}\leq k.$$

The detailed procedure of solving joint sparse representation coefficients $\hat{W}$ is presented in Section 3.3.

### 3.3. MBCS Technique for Solving Joint Sparse Representation Coefficients

In this study, the Multitask Bayesian Compressive Sensing (MBCS) technique is applied to solve the joint sparse representation coefficients W in Section 2.2.2 [48], in order to capture more of the temporal–spatial correlation information regarding gait. Due to multi-sensor gait data that are contaminated by noise, the test gait Y can be represented as Y=AW+E, i.e.,

(19) $$\left[\begin{array}{l} y_{1}\\ y_{2}\\ \vdots \\ y_{J} \end{array}\right]=\left[\begin{array}{l} A_{1}\\ A_{2}\\ \vdots \\ A_{J} \end{array}\right][W_{1},W_{2},\cdots ,W_{J}]+\left[\begin{array}{l} e_{1}\\ e_{2}\\ \vdots \\ e_{J} \end{array}\right]$$

where $E={\left[e_{1},e_{2},\cdots ,e_{J}\right]}^{T}$ denotes the noise. Here, the noise $e_{j}$ satisfies a Gaussian distribution with an unknown parameter $\beta_{0}$, and the prior distribution function of $y_{j}$ is defined as

(20) $$p(y_{j}|W_{j},\beta_{0})={\left(\beta_{0}/2\pi \right)}^{N/2}\exp(-\frac{\beta_{0}}{2}||y_{j}-A_{j}W_{j}|{|}_{2}^{2}).$$

Then, the prior distribution function of the sparse coefficients $W_{j}$ is defined as

(21) $$p(W_{j}|\beta )=\prod_{i=k}^{M}N(W_{j,k}|0,\beta_{k}^{-1})$$

where $W_{j,k}$ denotes the kth sparse representation coefficient of sensor j and $N(\cdot |0,\beta_{k}^{-1})$ refers to the Gaussian distribution function whose mean is equal to zero and variance $\sigma^{2}=1/\beta_{k}$.

After the optimal parameters $\beta ,\beta_{0}$ are estimated by constructing a likelihood function, we can solve the joint sparse representation coefficient ${\left\{W_{j}\right\}}_{j=1}^{J}$. The production of the solution for ${\left\{W_{j}\right\}}_{j=1}^{J}$ is presented in the Appendix A, and the detailed procedure for the solution can be found in reference [48].

### 3.4. The Definition of Minimal Residual Error Rule for Gait Classification

Based on the estimated solutions of the joint sparse representation coefficients $\hat{W}$, we firstly define the matrix indication function $\delta_{i}(\cdot )$ whose entries are zero except for those associated with gait class i. i.e.,

(22) $$\delta_{i}(\hat{W})={\left[0^{T},\cdots ,0^{T},W_{i}^{T},0^{T}\cdots 0^{T}\right]}^{T}.$$

Then, the approximation of test gait sample Y can be defined as

(23) $$\tilde{Y}=A\delta_{i}\left(\hat{W}\right).$$

The minimal residual error between Y and $\tilde{Y}$ can be defined as

(24) $$\underset{i}{\min}\;r_{i}\left(Y\right)={\left\|Y-A\delta_{i}\left(\hat{W}\right)\right\|}_{2}.$$

Thus, the class label of test gait Y can be identified by the following minimal residual error rule [23,33,41]:

(25) $$\hat{i}=\underset{i}{\arg\min}{\left\|Y-A\delta_{i}(\hat{W})\right\|}_{2}^{2},i=1,2,\cdots ,L.$$

## 4. Materials and Methods

### 4.1. The Selection of Multi-Sensor Gait Data

In this study, the multi-sensor gait data were selected from an open wearable action recognition database (WARD) database from University of California, in order to evaluate the feasibility of our proposed technique. In the selected WARD database, the multi-sensor gait data with different walking patterns are sampled from a total of 20 subjects (7 female subjects and 13 male subjects) with ages ranging from 19 to 75 years. All participants had no known injuries or abnormalities that could affect their walking pattern. The wearable motion sensor networks consist of five wireless motion sensors (two are located on the wrists, one is located on the waist, and two are positioned on the ankles, all using elastic belts). Each sensor node integrates a 3-axis (i.e., *x*-axis, *y*-axis and *z*-axis) accelerometer with a range of ±2 g and a 2-axis gyroscope with a range of ±500 dps, and each axis is reported as a 12 bit value to the sensor node. Each subject is asked to perform 13 activity or gait patterns such as stand, sit, lie down, walk forward, walk left-circle, walk right-circle, turn left, turn right, go upstairs, go downstairs, jog, jump, and push a wheelchair. Each subject is asked to perform each pattern 5 times. The sample ratio is set to 20 Hz. More details about the multi-sensor data collection are available at the website (http://www.eecs.berkeley.edu/~yang/software/WAR/).

### 4.2. Data Preprocessing

In this study, in order to effectively validate the feasibility of our proposed method, we randomly selected nine gait or activity pattern classes from all 13 gait patterns, such as stand, sit, walk forward, turn left, turn right, go upstairs, go downstairs, jog, jump. Firstly, the denoising for the recorded multi-sensor data is performed by using a fifth-order Butterworth with a low-pass filter at 30 Hz. Then, a sliding window technique is employed to segment the acquired time-sequence data into a sequence of fixed-length windows, in order to gain valuable correlation information from the collected multi-sensor gait data [16,24]. Here, the window is set to a length of N=200 with 50% overlap, and 10 windows were randomly extracted from each subject’s data. Here, each subject’s data consist of the 9 different gait pattern classes data, each class including 10 windows’ data (i.e., each subject’s data includes 10 stand data, 10 sit data, 10 walk forward data, 10 turn left data, 10 turn right data, 10 go upstairs data, 10 go downstairs data, 10 jog data, 10 jump data). In this study, the 20 subjects’ total data are used to train and test, in order to objectively evaluate the classification performance. The total sample data size is 1800 (i.e., 20 subjects × 9 gait pattern classes × 10 window samples), as shown in Table 1. In addition, we perform the classification task of nine gait patterns (walk forward, walk left, walk right, go upstairs, go downstairs, run forward, sitting), in order to further evaluate the feasibility of our proposed model for multi-sensor gait telemonitoring applications.

### 4.3. Training and Testing the Gait-Classification Model

In this work, the ten-fold cross validation strategy is adopted to objectively and accurately evaluate gait-classification performance [13,14,15]. Here, all 20 subjects’ data are divided into ten subsets, each including 2 subjects’ data. To effectively evaluate the generalization performance, the data that are used to test are not included in the training samples data. The detailed training and testing procedure is as follows: Firstly, the training set is constructed to develop a gait-classification model. Here, 18 subjects’ data are randomly selected as the training set, containing a sample size of 1620 (i.e., 180 stand data, 180 sit data, 180 walk forward data, 180 turn left data, 180 turn right data, 180 go upstairs data, 180 go downstairs data, 180 jog data, 180 jump data). In the process of training the classification model, a nine-fold cross-validation scheme is employed due to the limited data size. That is, all selected 18 subjects’ data are randomly divided into nine subsets, each subset including 2 subjects’ data. Eight subsets are arbitrarily selected as the training set while the remaining one is used to test. The above steps are repeated until each subset is employed to test. After the classification model is yielded, the remaining two subjects’ data that are not included in training set are used to test the generalization ability. Each subject’s data must be used to test the generalization ability. Finally, the whole averaged classification result is obtained for all subjects.

### 4.4. Evaluation Criteria for the Proposed Technique

In addition, some common criteria are adopted to objectively assess the performance of the proposed gait-classification model using multi-sensor gait data, and they are respectively defined as follows.

(1) Compression ratio (CR)

The compression ratio is used to quantitatively evaluate the ability to compress the multi-sensor gait data, and is defined as follows:

(26) $$CR=\frac{N-M}{N}\times 100\%$$

where N and M denote the original data length and the compressed data length, respectively.

(2) Accuracy

Accuracy is employed to objectively evaluate the classification performance, and it is defined as the ratio of the number of gait patterns accurately identified to the total of gait patterns in the tests.

(27) $$Accuracy=\frac{NA}{NT}\times 100\%$$

where NA and NT denote the number of gait patterns accurately identified and the total number of gait patterns, respectively.

(3) Precision

Precision is defined as a measure of the ability of the classifier to detect gait patterns with class i in the confusion table.

(28) $$Precision=\frac{NP}{NP+NF}\times 100\%$$

where NP and NF denote the number of gait patterns accurately identified as class i and class j, respectively.

(4) Recall

Recall is defined as a measure of the ability of the classifier to specify gait patterns with class j in the confusion table.

(29) $$Recall=\frac{NF}{NP+NF}\times 100\%.$$

In addition, in this study, statistical analyses to determine the mean and standard deviation (SD) are performed for the outcomes obtained by the different classification algorithms. All indices used are tested by means of a statistical analysis such as ANOVAs, in order to further test the objectivity of the outcomes obtained by the different classification algorithms. Here, all different algorithms that are considered as independent variables are performed on all indices.

In this study, all algorithm programs are developed in Matlab 2013a, and they are performed on a computer with an Intel(R) Core(TM) i5-3470 3.20GHz CPU, 4.00GB RAM, and Windows 7 operating system.

## 5. Results

### 5.1. The Evaluation of the Effect of DCS on the Developed Neighboring JSRC

Firstly, we evaluate the effect of the simultaneous compression of multi-sensor gait data by DCS on our developed neighboring JSRC(SRCC-JSRC) model. For comparison, we also perform a JSRC model and a KNN-based JSRC(KNN-JSRC) model. For DCS, the sparse binary matrix was selected as the random projection matrix where only two nonzero entries are included in each column. All 20 subjects’ data are used to train and test, and the ten-fold cross validation strategy is adopted to objectively evaluate gait-classification performance. Firstly, we compared three JSRC-based classifications’ performances based on multi-sensor gait data which are simultaneously compressed by DCS. The number of neighboring samples are selected as 20, 40 and 60, respectively. All comparative results are shown in Table 2. From Table 2, we can observe that when the number of neighboring samples is selected as 40 or 60, all neighboring sample-based JSRC models can produce a better accuracy of 94% as well as a lower computational time cost when compared with the JSRC model. These results suggest that the simultaneous compression of multi-sensor gait data by DCS can provide the valuable high-correlation information regarding gait for the JSRC model.

### 5.2. The Assessment of Gait-Classification Performance Based on the Different Compression Ratios

**Figure 2:**
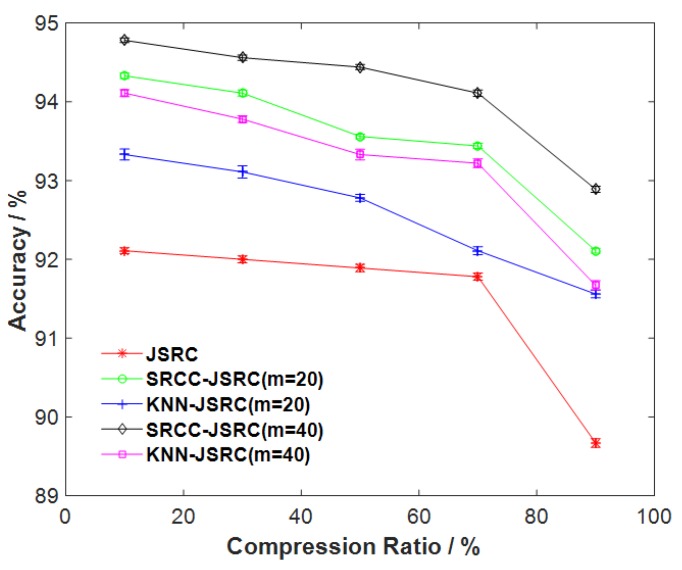
Comparative results of the different gait-classification performances based on the different compression ratios.

Next, we evaluate the classification performance of our proposed method based on the different compression ratios to examine the feasibility of the energy-efficient telemonitoring of gait. The number of neighboring samples are selected as 20 and 40, respectively. The comparative results from all JSRC-based models are shown in Figure 2. The errors of accuracy based on the different compression ratios are also presented in Figure 2. Table 3 shows the mean and SD values of the best accuracy from all selected algorithms. As shown in Figure 2 and Table 3, the SD of all accuracies can obtain lower error. From Figure 2, we obviously see that, based on the different compression ratios, our proposed method (SRCC-JSRC) can produce better accuracy than KNN-JSRC and JSRC, suggesting that the neighboring samples by our defined criteria contain the more valuable information about the test sample. In particular, when the compression ratio increases from 10 to 70%, SRCC-JSRC (m=40) reaches the best accuracy. These results showed that our proposed method can yield the superior classification performance when the compression ratio is properly selected. That is, our proposed method can potentially produce the best classification performance as well as the best energy efficiency of sensors. In addition, from Figure 2, we also find that all JSRC-based gait classifications produce poor accuracy when the compression ratio is more than 70%. This suggests that the higher compression ratios could lose the more valuable correlation information regarding gait.

**Figure 3:**
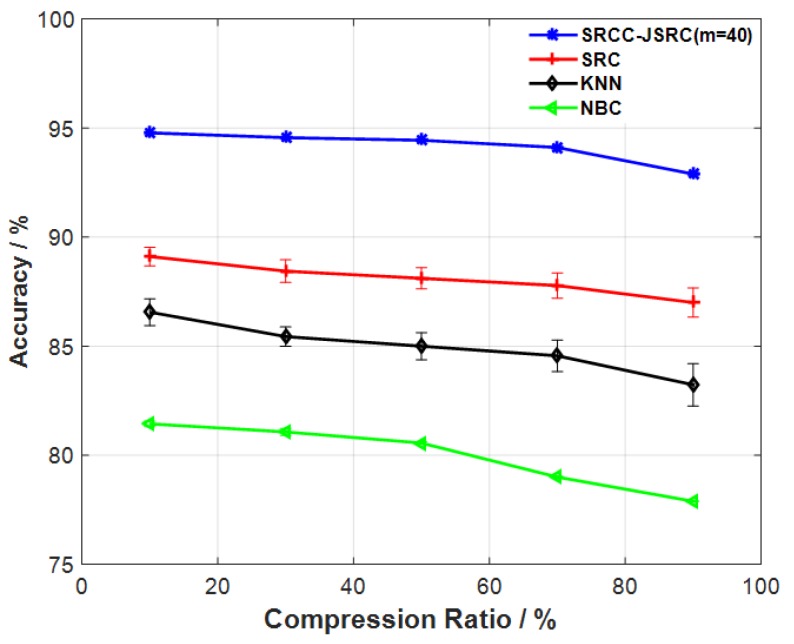
Comparison of accuracy of our proposed technique with KNN, naive Bayesian classifier (NBC), and sparse representation classification (SRC).

In addition, we also compared our proposed model to some tradition classification models such as SRC, naive Bayesian classifier (NBC), and KNN models. Here, the number of nearest neighboring samples are selected as 40 (i.e., m=40). The comparative results are shown in Figure 3. As shown in Figure 3, our proposed model significantly outcompetes SRC, NBC, and KNN models. In this comparison, the NBC technique is the poorest. These results demonstrate that our proposed model can gain more distinctive temporal–spatial correlation information associated with gait pattern change.

### 5.3. The Evaluation of Computation Time Cost Based on the Different Compression Ratios 

**Figure 4:**
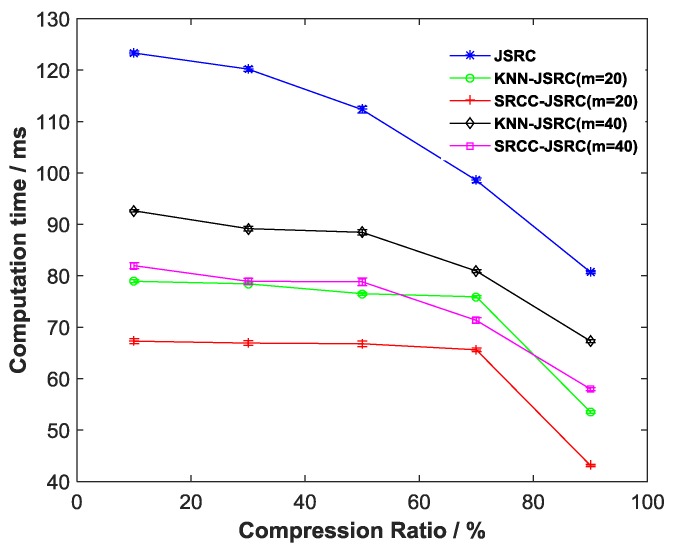
Comparative results of computation time from the different JSRC-based gait-classification algorithms.

Meanwhile, we also evaluate the computational time cost of all JSRC-based models based on the different compression ratios. Here, statistical analyses to determine the mean and standard deviation (SD) are performed for the obtained computational time from the different classification algorithms. All comparative results are shown in Figure 4. The errors of the obtained computational time based on the different compression ratios are also presented in Figure 4. Table 4 shows the mean and SD values of the lowest computation time from all selected algorithms. As shown in Figure 4 and Table 4, the SD of all computation times can obtain lower error. From Figure 4, we can obviously see that the computation time cost of each JSRC-based model is reduced as the compression ratio increases. In comparison, our proposed method (SRCC-JSRC) has the lowest computational time cost. In particular, the lowest computational time cost of SRCC-JSRC (m=20) remains only 67 ms while the compression ratio increases from 10 to 70%. These results demonstrate that our proposed method could possibly result in a lower computational time cost and better energy efficiency of sensors when the compression ratio is properly selected. Besides this, we also observe that, once the compression ratios are more than 70, all computation times significantly decline. This is because the higher compression ratios could yield fewer multi-sensor gait data to be processed.

### 5.4. Evaluation of Our Proposed Model for Gait Telemonitoring 

We perform the task of classification for nine gait patterns (walk forward, walk left, walk right, go upstairs, go downstairs, run forward, sitting, jog, jump), in order to further evaluate the feasibility of our proposed model for multi-sensor gait telemonitoring applications. Here, the number of nearest neighboring samples is selected as 40, and the compression ratio is set to 50%. Table 5 shows the confusion table for the classification of the nine gait patterns. In Table 4, the entry in the ith row and jth column refers to the count of gait patterns belonging to class i that are classified as class j. As shown in Table 5, the whole averaged classification accuracy across all nine gait patterns reaches 95%, suggesting that our proposed technique can accurately classify these nine gait patterns. In terms of individual patterns, the “turn left” and “jump” patterns obtain the maximal value of 100% and the “jog” pattern gains the best precision value of 100%. The “downstairs” pattern gains the same maximal precision and recall value of 97%, and the “sitting” pattern obtains poor precision and recall values. For the “jump” pattern, its precision is equal to 100%, whereas its recall is only 90%. The possible reason for this is that some samples of “jump” are easily misclassified as “walk forward” and “downstairs”, but samples of “walk forward” are not misclassified as “jump”. In conclusion, these results show that our proposed technique can capture the more distinctive and high-correlation information associated with gait for multi-sensor gait classification. Our proposed method has great potential for gait telemonitoring applications. 

In addition, the results of statistical tests based on ANOVAs are presented in Table 6. As shown in Table 6, all significance values are less than the significance level value of 0.05, suggesting that the significance of the difference among classification algorithms is at an acceptable level. This further validates the objectivity of the outcomes obtained by the different classification algorithms. 

## 6. Discussion

The results of present studies demonstrate that the hybrid technique of DCS and JSRC can take advantage of data joint sparsity to jointly process multi-sensor gait data, which can capture the more valuable high-correlation information hidden in multi-sensor gait data. This greatly contributes to the best classification performance as well as to the energy efficiency of sensors in WBANs-based gait telemonitoring applications. Currently, the discovery of powerful techniques for the quantitative analysis of multi-sensor gait data has become a research focus in the gait telemonitoring field [10,11,12]. One important issue is how to gain the higher-correlation information regarding gait for the best gait-classification performance as well as for improving the energy efficiency of sensors [13,26,27,28].

In the present studies, considering that both DCS and JSRC have the best ability to jointly process multi-signals based on data joint sparsity, we investigated the feasibility of the hybrid technique of DCS and JSRC for gait telemonitoring by jointly processing multi-sensor gait data [32,33,34]. In particular, we try to take advantage of the joint sparsity of multi-sensor gait data to develop a novel neighboring JSRC model for the best classification performance as well as lower computation time. In this study, we firstly evaluate the effect of the simultaneous compression of multi-sensor data by DCS on the JSRC-based classification model. As illustrated in Table 2, all JSRC-based gait-classification models can yield better accuracy. This suggests that the DCS technique has the superior ability to capture the higher-correlation information regarding gait by jointly compressing multi-sensor gait data, and all compressed multi-sensor gait data containing the more valuable information significantly helps to produce the better classification performance of the JSRC-based model. These results further suggest that it is feasible that DCS and JSRC can jointly process multi-sensor gait data for gait classification based on data joint sparsity.

Next, we evaluate the feasibility of our proposed method for multi-sensor gait classification based on the different compression ratios. As shown in Figure 2, all selected JSRC models can yield a better gait-classification performance when compression ratios are properly chosen. In the comparison, our proposed model is best. This suggests that our proposed model potentially yields the best performance as well as the best energy efficiency of sensors. In addition, we also compared our proposed method to the traditional SRC, NBC, and KNN models. As shown in Figure 3, our proposed method significantly outcompetes the selected traditional methods. A possible reason for this is that the selected neighboring training samples can contain the more distinctive and high-correlation information associated with gait, which significantly improves multi-sensor gait-classification performance. However, the traditional SRC model does not gain the valuable high-correlation information as it does not jointly processes multi-sensors gait data [15,16,24,25]. Similar results in a study on hyperspectral image classification have been found in [45]. Besides this, from Figure 2, we also find that all JSRC-based gait-classification models result in poor accuracy when the compression ratios are more than 70%. This is because the compressed multi-sensor gait data possibly contains more redundant information, thus destroying the classification performance. Similar results in studies on gait telemonitoring have been also reported in [10,15,16,29]. 

In addition, we also evaluate the computational time cost corresponding to all JSRC-based models based on the different compression ratios. As illustrated in Figure 4, in comparison, our proposed method has the lowest computational cost. The main reasons for this are that the proposed novel neighboring JSRC algorithm has a maximum time complexity of only $T_{1}+O(w^{2}m)$ ($T_{1}$ denotes the time for searching the m nearest neighbor samples) to produce the best classification performance. However, the traditional JSRC algorithm has a computational complexity of $O(w^{2}n)$ (n≥m, n is the total number of training sample data points) for multi-sensor gait performance [33,41,43]. In conclusion, all the above results show that our proposed technique has the potential ability to gain the best classification performance, lower computational time cost, and better energy efficiency of sensors in multi-sensor gait classification.

In this study, our proposed model is also employed to classify nine different multi-sensor gait patterns, in order to further examine the practicality of gait telemonitoring applications. As shown in Table 5, our proposed technique shows high-quality gait-classification performance. This suggests that our proposed technique may enforce the robustness of coefficient estimation, which helps to exactly solve the joint sparse representation coefficients. In particular, this is because our hybrid technique can take advantage of the joint sparsity of multi-sensor gait data to gain the more distinctive information associated with the spatial, temporal, and dynamic correlations of gait [13,26,27,28], which greatly contribute to identifying multi-sensor gait pattern change. Similar findings have been reported in [9,12,46].

## 7. Conclusions

In this study, an advanced hybrid technique of the DCS and JSRC models is proposed for multi-sensor gait telemonitoring. In our technique, both DCS and JSRC feasibly take advantage of data joint sparsity to jointly process multi-sensor gait data. All multi-sensor gait data jointly compressed by DCS contain the more valuable high-correlation information related to gait, and are directly used to develop the JSRC model. This can avoid the multi-sensor gait data reconstruction step and potentially increase the energy efficiency of the sensors. A novel neighboring JRSC model can be developed to gain the best gait-classification performance as well as a lower computational time. It is very hopeful that our proposed technique can serve as a powerful tool for processing multi-sensor gait dat in gait telemonitoring applications. Future research paths are to find the effective methods that can significantly improve the classification performance of multi-sensor gait data while compression ratios are higher.

## Figures and Tables

**Table 1:** The total amount of available sample data.

All Samples Data Size		
All Subjects	Gait Pattern Classes	Window Samples
20	9	10

**Table 2:** Comparative results from the different JSRC-based gait-classification performance.

Classification Algorithms	Accuracy/%	Computation Time/ms
JSRC	91.33	132.8510
KNN-JSRC (*m* = 20)	94.00	77.0239
KNN-JSRC (*m* = 40)	94.11	87.4365
KNN-JSRC (*m* = 60)	94.44	102.5404
SRCC-JSRC (*m* = 20)	92.67	66.1759
SRCC-JSRC (*m* = 40)	94.44	78.0513
SRCC-JSRC (*m* = 60)	94.89	90.7440

It is noted that KNN is the abbreviation of k-nearest neighbor and SRCC is the abbreviation of sparse representation coefficients-inducing criterion.

**Table 3:** The statistical results the best accuracy from all selected algorithms.

Algorithms	SRCC-JSRC (*m* = 40)	SRCC-JSRC (*m* = 20)	KNN-JSRC (*m* = 40)	KNN-JSRC (*m* = 20)	JSRC
Mean value (%)	94.58	94.23	93.31	93.13	92.11
SD value	(0.043, 0.076)	(0.029, 0.038)	(0.041, 0.066)	(0.042, 0.078)	(0.037, 0.054)

**Table 4:** The statistical results for the lowest computation time from all selected algorithms.

Algorithms	SRCC-JSRC (*m* = 40)	SRCC-JSRC (*m* = 20)	KNN-JSRC (*m* = 40)	KNN-JSRC (*m* = 20)	JSRC
Mean value (ms)	77.65	65.98	88.03	76.45	132.06
SD value	(0.032, 0.065)	(0.025, 0.034)	(0.036, 0.061)	(0.036, 0.072)	(0.032, 0.049)

**Table 5:** The confusion table for classification of the nine different gait pattern classes.

	Standing	Sitting	Walk Forward	Turn Left	Turn Right	Upstairs	Downstairs	Jog	Jump	Total	Recall
Standing	186	14	0	0	0	0	0	0	0	200	93%
Sitting	6	184	10	0	0	0	0	0	0	200	92%
Walk forward	4	0	194	0	0	2	0	0	0	200	97%
Turn left	4	0	6	188	2	0	0	0	0	200	94%
Turn right	6	4	0	0	190	0	0	0	0	200	95%
Upstairs	0	6	0	0	0	196	0	4	0	200	98%
Downstairs	4	2	0	0	0	0	194	0	0	200	97%
Jog	0	0	0	0	0	4	1	200	0	200	100%
Jump	0	0	6	0	0	4	6	4	180	200	90%
Total	210	212	216	188	192	206	201	208	180		
Precision	88%	88%	90%	100%	99%	96%	97%	96%	100%		

**Table 6:** The results of statistical tests for all indices based on ANOVAs.

Indices	Compression Ratio	Accuracy	Computation Time	Precision	Recall
*F* values	70.51	101.21	146.81	224.92	182.47
Significance values	0.0235	0.0379	0.0342	0.0288	0.0357

## Appendix A

In the MBCS technique, the set of unknown parameters $\beta ={\left\{\beta_{k}\right\}}_{k=1,2,\cdots ,N}$ is the hyper-parameter for the prior distribution of $W_{j}$. Here, in order to obtain the best sparsity of coefficients $W_{j}$, the hyper-parameters $\beta_{0},\beta$ are assumed to satisfy a Gamma distribution, and the prior probabilities of $\beta_{0},\beta$ are defined, respectively, as

(A1) $$p(\beta_{0}|a,b)=Ga(\beta_{0}|a,b),$$

(A2) $$p(\beta |c,d)=\prod_{k=1}^{M}Ga(\beta_{k}|c,d),$$

where a,b,c, and d, respectively, denote four different parameters in the Gamma distribution. When a=b=c=d=0, the super prior probabilities of $\beta_{0},\beta$ are available. So, the following maximum likelihood function of $\beta_{0},\beta$ is defined as

(A3) $$\{\beta ,\beta_{0}\}=\arg\min\sum_{j=1}^{J}\log\int dW_{j}p(y_{j}|W_{j},\beta_{0})p(y_{j}|\beta ).$$

Once $\beta_{0},\beta$ are solved, the posterior probability distribution of $W_{j}$ is defined as

(A4) $$p(W_{j}|y_{j},\beta ,\beta_{0})=N(W_{j}|\mu_{j},\Sigma_{j})$$

where $\mu_{j}=\beta_{0}\Sigma_{j}{\left(A_{j}\right)}^{T}y_{j}$ is the mean, $\Sigma_{j}={\left[\beta_{0}{\left(A_{j}\right)}^{T}{\tilde{A}}_{j}+\Gamma \right]}^{-1}$ is the covariance, and $\Gamma =\mathrm{diag}(\beta_{1},\beta_{2},\cdots ,\beta_{N})$ is the diagonal matrix.

In order to effectively estimate the parameters $\beta_{0},\beta$, the likelihood function is constructed as follows:

(A5) $$L(\beta ,\beta_{0})=\log p(y_{j}|\beta ,\beta_{0})\;=-\frac{1}{2}\sum_{j=1}^{J}\left(N\log2\pi +\log|C_{j}|+y_{j}C_{j}^{-1}y_{j}\right)$$

where $C_{j}=[\beta_{0}^{-1}I+A_{j}\Gamma^{-1}{\left(A_{j}\right)}^{T}]$. Usually, the derivative of $L(\beta ,\beta_{0})$ to $\beta ,\beta_{0}$ is assumed to be zero, and the iterative rules of $\beta ,\beta_{0}$ are respectively defined as follows:

(A6) $$\beta^{new}=\frac{J-\beta_{k}\sum_{k=1}^{J}\Sigma_{j,(k,k)}}{\sum_{j=1}^{J}\mu_{j,k}^{2}},$$

(A7) $$\beta_{0}^{new}=\frac{\sum_{k=1}^{J}\left(M-N+\sum_{k=1}^{M}\beta_{j}\Sigma_{j,(k,k)}\right)}{\sum_{j=1}^{J}{\left||y_{j}-A_{j}\mu_{j}|\right|}_{2}^{2}}.$$

When the optimal parameters $\beta ,\beta_{0}$ are estimated, the solutions of the joint sparse representation coefficients ${\left\{W_{j}\right\}}_{j=1}^{J}={\left\{\mu_{j}\right\}}_{j=1}^{J}$ are available.
